# Hygienic Condition of Towns

**Published:** 1845-01

**Authors:** 


*Hygienic Condition of Towns.—Over-Labor and Late Hours of Business.*—It has long been a fact well known to the profession that towns are peculiarly inimical to the prolongation of life; that a very much smaller proportion of their inhabitants reach ripe old age than of those whose lives are spent in agricultural districts. Until within the last few years, however, this generally-acknowledged *truth* had not become an undeniable fact, susceptible of being palpably demonstrated by figures. Thanks to the elaborate statistical investigation which are being carried on in every part of England, we are now able to ascertain with the greatest precision, not only the comparative salubrity of town and country, but also the different salubrity of towns compared with each other. Thus, in the last report of the registrar-general of births, deaths, and marriages, in England, we find a comparative tabular view of the mortality at different ages in the county of Surrey, and in the towns of Liverpool and London. Taking, at the moment of birth, a generation of one hundred thousand, it appears that in Surrey fifty thousand (half) would reach the age of fifty; twenty thousand the age of seventy-five; and ten thousand the age of eighty. In London, forty thousand would reach the age of fifty; ten thousand the age of seventy-five; and five thousand the age of eighty. In Liverpool, twenty-five thousand, only, would reach the age of fifty; five thousand, only, the age of seventy-five; and one thousand five hundred the age of eighty.

The immense disparity in the duration of life in these different localities is no subject of surprise to the well informed medical hygienist. In it he sees the operation of known causes, which the ignorance of mankind has accumulated, and which it is his duty to point out. Among those causes, there is one which, no doubt, exercises the most pernicious influence over the health and longevity of the denizens of towns, and which has, of late, excited the attention of the public to a great extent, viz., over-labor, and lengthened in-door confinement.

For many years the sympathies of the public generally have been justly given to the unfortunate females and children whose bodily strength is tasked to such an inhuman extent in the factories of our large commercial districts, and we are now pleased to find that philanthropists are becoming alive to the important fact that it is not only the factory laborers whose health and strength requires protection. There are many other classes of society to whom it is equally necessary. Indeed, this feeling is so rapidly gaining ground that we are on the eve of important changes in the social organization of labor, wherever large masses of people are congregated. True it is that Lord Ashley's amendment with respect to factory labor, which may be said to have embodied this feeling, in our legislature, has been definitely rejected by an overwhelming majority; in that rejection, however, we do not see the overthrow of a principle, but merely the irresistible sway of economical and political considerations. Under the influence of the first charitable impulse, the House of Commons decided that protection should be given to the young, and thus spontaneously acknowledged the sacred rights of humanity; but the sacrifice was too great, too momentous, to be permanent. Although ourselves convinced of the *absolute necessity* of protecting children and women in their struggle with machinery and commercial rivalry, and inclined to believe that the pecuniary loss to themselves and their employers has been greatly exaggerated, yet we admit the possibility of the change being attended, as most changes are, with immediate painful consequences to all parties.

We have, however, greater confidence than our opponents, most of whom we believe to be conscientious in the defence of their opinions, in the elasticity of the laws regulating labor. We cannot bring ourselves to believe that an hour or two additional taken from the daily work of the children and women of our factory districts would entail destruction on our foreign and domestic commerce. Moreover, it must be evident to any one that if, as the manufacturers assert, their profits depend on the eleventh and twelfth hours of child labor, their position would be the same were they to reduce wages a sixth and take on the additional hands. Now, miserably small as are the wages at present, inadequate, as the remuneration undoubtedly is, for the labor performed, yet we had rather see the pay still further reduced than allow women and children to be worked from 6 o'clock in the morning until 8 in the evening, including meals. As physiologists and practitioners, we cannot be blind to the fact that such labor is calculated to sap the very sources of life, and to lay the foundation for the cachectic diseases which, in reality, do carry off the successive generations of factory laborers, as is evident from the statistics we have alluded to. There is, alsc, another important feature in the case, which must not be lost sight of, which is, that the operation of machine.ry has displaced the operation of the laws of nature. It is the father of the family who ought to provide for his wife and children, and not the wife and children who ought to provide for the father, as is now the case. When the working of society produces such anomalies as these, it is the duty of the legislature to interfere, and to save, even at the expense of enormous sacrifices to the country at large, women and children from being ground to the earth by over-labor, to prevent the population of manufacturing towns from being poisoned at its very source. Such interference is merely giving to the weak and the young the protection which humanity renders it imperative on the legislators of every country to afford; it is not, properly speaking, interfering with the laws of labor. The government of a nation has, however, in our opinion, a *right* to interfere, even with the labor of adult men, on hygienic national grounds.

The ten-hours' factory bill will, no doubt, be again brought in the House, perhaps under more favorable circumstances, and we feel convinced that before many years have elapsed it will be the law ot the land. Before, however, we dismiss the subject for the present, we must remind our medical brethren that they are, to a certain extent, the guardians of the public health—that on all general sanatory questions their opinion has great weight—that it is, consequently, their duty to prepare for future debates by collecting as much evidence as they can possibly bring to bear on the subject. In the meetings which have taken place in the manufacturing towns of the north of England in favor of the ten-hour clause, we have observed among those who have taken an active part in the proceedings many medical practitioners of weight and standing, whose statements have every where been received with respect and attention. Their example must not be lost to the profession, which we know will continue to show itself, as in all other great social questions, the true friend of the suffering and afflicted.

In the meantime there are others among the denizens of towns scarcely less afflicted than the factory artisans, and whose sufferings are beginning to attract public attention. Few of these are more deserving of commiseration than the young men and women who are employed in the shops of the metropolis, and of large towns generally. Obliged to rise early in order to prepare for the business of the day, they remain on their feet until late in the evening, without a respite. Their meals, even, are hurriedly despatched in the course of a few minutes, in order that they may not interfere with the claims of business. An important section of the metropolitan tradespeople—the drapers—have, we are pleased to see, taken up the subject with great spirit. Last year they offered a prize of twenty guineas for the best essay on "the evils of the present protracted hours of trade generally." This prize has been awarded to Mr. Thomas Davies, himself one of the sufferers, for a very able and elaborate essay on the subject. The essay of Mr. Davies reflects great credit on his instruction and abilities, and gives a vivid picture of the pernicious effects, both as to health, morals, and mental cultivation, of the discipline to which he and his associates have to submit in linen-drapers' shops. The day is consumed in the incessant repetition of services which, though slight, becomes trying to the strength, from their continuance. The assistant is not allowed to sit down, because it would be unbusiness-like, and when at last, at 9 or 10 o'clock, the shop closes to the public, he has still one, two, or three hours' work to perform, in order to fold and replace the various articles that have been disarranged during the day.

The result of this close confinement during the day and evening in a heated atmosphere, and of incessant labor, continued during a period of fourteen or sixteen hours, is very destructive to health, as might be anticipated. Many youths are obliged to return to their friends within a few months of their arrival at the establishments. Many others lose their previously robust health, and either die consumptive, or become confirmed invalids. But we refer our readers to the pamphlet of Mr. Davies for ample details on this interesting subject.

The evils are not confined to the linen-drapers. They apply with equal force to all tradespeople who open early and shut late, passing their lives in their shops or their back parlors. All medical gentlemen practising in large towns are unanimous in their opinion that the resident tradespeople, and their assistants, are among the most unhealthy part of the population, and that this unhealthiness is in a great measure attributable to the confinement which their occupations entail upon them. The evil is great, no doubt, but the question which naturally presents itself to us is—can it be remedied? We think that it certainly may be, and that without entailing upon the community any of the loss which, it is believed, by so many, would follow the adoption of the ten hours' factory clause. Indeed, the good sense of the sufferers themselves has pointed out the mode of relief. In London, and in several of our large provincial towns, associations have been formed for promoting the early closing of shops. These associations have every where received the support of philanthropists of wealth and station; and we may mention, that in Liverpool it is presided over by the mayor, and patronised by most of the influential residents.

By such means a certain amount of good may be effected. The wealthier tradespeople who are alive to the injurious effects of the present system on themselves and their subordinates will, no doubt, gladly avail themselves of the sanction of the affluent and influential to close their establishments at an early hour. But there will always be many needy or avaricious persons who will keep their shops open, were it only to profit by the closure of those of their neighbors, and thus will the end in view be partly defeated.

Such being the case, why should not the legislature take the subject in hand, and render it imperative on the part of all retailers to close their shops at a certain hour, say 6 or 7, P. M.? By the same rule that all the wholesale commerce of the country is carried on in the middle of the day, so might also the retail. The banks, boards, and offices of all description, close at an early hour, why should not retail establishments? Any inconvenience which might be felt would only be temporary. The habits of the community, of the poor as well as of the rich, would soon bend to the new hours of trade. In working out such an idea for practical purposes, many points would necessarily have to be taken into consideration. It might, for instance, be found necessary to except for certain periods, or on certain days, shops kept for the sale of provisions, &c. We merely suggest the principle as one which it would be easy to apply, and the application of which would materially improve the physical and moral condition of a very large and important class of the community. Such interference with labor as this, is not only allowable, but absolutely necessary, if we wish to make our improved knowledge of the laws which regulate health and disease conducive to the prosperity and welfare of mankind.—*Lancet.*

